# Effect of different teaching/learning approaches using virtual patients on student’s situational interest and cognitive load: a comparative study

**DOI:** 10.1186/s12909-022-03831-8

**Published:** 2022-11-07

**Authors:** Sura Ali Fuoad, Walid El-Sayed, Hesham Marei

**Affiliations:** 1 Department of Diagnostic and Surgical Dental Sciences, College of Dentistry, Gulf Medical University, Ajman, United Arab Emirates; 2 Department of Basic Medical and Dental Sciences, College of Dentistry, Gulf Medical University, PO Box 4184, Ajman, UAE; 3 Department of Oral Biology- College of Dentistry, Suez Canal Univesity, Ismailia, Egypt

**Keywords:** Virtual patient, Situational interest, Cognitive load

## Abstract

**Background:**

Virtual Patients (VPs) have been receiving considerable attention in medical education as an authentic learning and teaching approach. The study aimed to evaluate the effect of using different approaches of conduction of virtual patients (VPs) on students’ cognitive load and situational interest.

**Methods:**

The study is an experimental study. Two different cohorts have participated during the academic year 2019/2020 and 2020/2021. The first cohort (Group 1) was exposed to a lecture followed by an independent VPs session, and the second cohort (Group 2) was exposed to a collaborative VPs session. The situational interest and Cognitive load were compared between the two groups. All sessions are about one topic related to maxillofacial trauma.

**Results:**

Findings showed that there was no significant difference between the median score of the situational interest at repeated time points during the Collaborative VPs (Group 2). However, in group 1, there was a significant difference between the median score of situational interest at repeated time points during independent VPs where the lowest score was found to be at the end of the session. Also, results showed that the collaborative VPs (Group 2) showed a high median score of situational interest than both lecture and independent VPs (Group 1). Furthermore, the study showed that there is no significant difference in the intrinsic cognitive load among the three sessions. However, the extraneous cognitive load was low in collaborative VPs (Group 2) than in both lecture and independent VPs sessions (Group 1).

**Conclusion:**

The use of VPs in a collaborative interactive learning activity is more effective than its use as an independent learning activity in enhancing students’ situational interest and reducing cognitive load. However, giving independent VPs after the lecture with the same topic is considered a limitation of the study as this can affect the situational interest of the students by filling their gab of knowledge.

**Supplementary Information:**

The online version contains supplementary material available at 10.1186/s12909-022-03831-8.

## Introduction

Virtual Patients (VPs) have been receiving considerable attention in medical education as an authentic learning and teaching approach. In addition, and due to advancements in the fields of virtual reality and artificial intelligence, more extensive applications of VPs in health profession education are expected. In the context of medical education, VPs can be defined as “an interactive computer simulation of a real-life clinical scenario for teaching, learning, and assessment” [1]. Generally, there are two major distinguished categories or designs of VPs which are the `problem-solving’ and the `narrative’ approaches [2, 3]. For teaching clinical reasoning and diagnosis purposes, the problem-solving design is preferable in which students have to collect a range of information, usually from menus of possible history questions, lab tests, and physical examinations, and make diagnostic and management decisions based on their findings. While the narrative design is concerned with cause-and-effect purpose. This includes programs that have an emphasis on decision making which results in various outcomes over time [2, 3]. Furthermore, VPs can be classified according to the interaction of the learner with the VPs into linear and branched tree designs. In linear design, the learner has to finish one stage (diagnosis) to move to the next stage (treatment planning) of the VP. However, in the branched tree design, the learner follows the consequence of the decision taken, as the outcome could be in one end or multiple ends [4].

Several studies have shown that VPs can offer curricula that are rich in authentic activities and full of a variety of problems that can be a very effective tool for knowledge acquisition and retention [3, 5, 6]. In addition, several studies focused on the importance and the effect of the sequence of VPs before or after other learning instructional methods to achieve maximum benefit for the learner. Marei et al. found that using VPs as collaborative learning after a lecture is an efficient instructional method that leads to better learning and knowledge transfers compared to collaborative VPs before lecture [5].

McCoy et al., 2016 stated that VPs increase student engagement in terms of flow, relevance, and interest [7]. The study was conducted in the context of simulated clinical decision-making for first-year medical school in the Southwest United States where VPs provided case scenarios that required rapid-fire group clinical decisions during a timed exercise [7]. The author attributed this to the fact that VPs is an active technology-based learning strategy, which offers a problem-solving approach that allows student choice, immediate feedback, and peer collaborative discussion, which in turn leads to more student engagement. Monitoring students’ engagement throughout learning activities is, therefore, relevant. One strategy to do so is to measure students’ situational interest.

Situational interest can be defined as “an immediate affective response to certain conditions and/or stimuli in the learning environment that focuses one’s attention on the task, which may or may not last over time” [8]. It has been shown to be a good predictor of students’ engagement with learning activities and learning outcomes [8]. Instructional activities that are challenging, novel, attracting for learner attention, offering the opportunity for exploration, and be enjoyable have been shown to be triggers of situational interest [9]. How situational interest evolves throughout a learning instruction, however, is a controverse topic. Hidi and Renninger in their work done in 2006 proposed the four-phase model beginning with a) triggering situational interest, b) maintaining situational interest, c) developing less or incomplete individual interest, and d) finally, well-developed or complete individual interest [10]. Rotgans and Schmidt, on the other hand, have observed that the main trigger of situational interest is a lack of knowledge to solve specific problems, therefore learners would develop high SI in the topic to fill the gap in their knowledge that is necessary to solve a specific problem. However, situational interest will go down as soon as the learner’s gap of knowledge is filled. Therefore, the development of interest in the content is a prerequisite for the maintenance of the SI [8, 11].

Interest and engagement in learning activities, however, are not the only factors that are relevant to students’ outcomes. Cognitive load can also influence learning outcomes. Cognitive load is managing the capacity of working memory, so it can process novel information to construct schemas in long-term memory [12]. Cognitive load can be classified into three types: intrinsic, extraneous, and germane [13]. Extraneous cognitive load is concerned with the instructional method used to present the educational material. While the intrinsic cognitive load is concerned with the inherent difficulty and complexity of the knowledge that needs to be acquired. Germane cognitive load is the remaining cognitive capacity of the working memory that is required to create or modify schemas while consciously processing information [14].

A study conducted by Park et, al., in 2015 using One hundred twenty seven collage undergraduate students enrolled in “Computer literacy” classes, suggested that a well-designed pedagogical instruction that contains multimedia promotes learning through enhancing situational interest without increasing cognitive load [15]. However, it is not yet clear what is the instructional design of such hybrid systems in which multimedia, narrative voice, and other effects are used to decrease the cognitive load while at the same time triggering and promoting situational interest. According to the interest learning theory, the situational interest (attention & motivation) of the student depends on two important factors: a) student characteristics (age, gender and ethnicity, goals, knowledge, and experience) and b) instructional variables (Teaching and learning environment) [10, 16].

However, there is still a need for more research about the effectiveness of different teaching/learning approaches using VPs regarding the cognitive load and situational interest of the students. Also, more research needs to be conducted to see the best way to use VPs to achieve the maximum benefits of their educational purpose whether it is used alone or in a collaborative session with other methods of teaching. Our study aimed to find an answer to the following research question “Would Situational Interest and cognitive load differ when a VPs session about maxillofacial trauma is conducted individually and preceded by a lecture about the same topic, compared to the same VPs session conducted as an interactive and collaborative session within the lecture?”

## Methods

The study design was an Experimental comparative study that was conducted at Gulf Medical Univesity -UAE. The study was reviewed and approved by the University Ethical Committee IRB Ref. no. RB/MHPE/STD/20/Dec-2020. Students were either exposed to a lecture followed by an independent VPs session (Group 1, n: 69) or to a single collaborative VPs session within a lecture (Group 2, n: 70). Lecture and VPs sessions were about the same topic. All the dental students were in their 4th academic year (semester 8) of a 5-year discipline-based dental program. During this academic year, students are exposed to the oral surgery discipline in which the study was conducted on one of its topic. Exclusion criteria: students who missed one of the two sessions and students who did not complete the full questionnaire.

### Virtual patients

For the teaching and learning sessions, one branching tree VPs designed with a single end node was used. In this type of design, multiple learner pathways are used which allows learners to choose between asking for a lab result or confirming the diagnosis and moving forward with the analysis. The learner pathway is based on the decisions taken by the learner at every strategic node [4]. The VPs are in the form of E-learning scorm package that is uploaded on Moodle and used by the instructor and learners. These VPs were based 3 clinical cases of real patient scenarios different types of maxillofacial injuries. Real patient radiographs, lab results, intra-oral photos, and records for other special investigations were used at different stages of the VPs path, while two-dimensional graphics (Two-dimensional graphics means using regular videos and image technology and not three-D technology) were used to represent different clinical settings and different characters within the VPs. The main purpose of VPs was to teach and learn problems related to maxillofacial trauma. Diagnosis and treatment planning of mandibular fractures were the main goals of the session. All the participants worked with the same cases.

### Procedure

The first cohort (Group1) received a lecture followed by a learning session using one independent VPs while the other cohort (Group 2) received the discussion-based session (collaborative session) in which VPs are a part of the contents of the interactive lecture. All interventions were delivered by the same subject expert.

1. **Lecture followed by independent VPs sessions (Group1):** All students received the same lecture which was about maxillofacial trauma in the oral and maxillofacial regions. The lecture was conducted for 40 minutes as a PowerPoint presentation. The lecture followed a teacher-centered approach and targeted the learning objectives mainly related to the diagnosis of specific conditions through identifying the relevant history, signs and symptoms, and the required investigations. After that students take 20 minutes’ rest and then they had VPs as an independent activity for 40 minutes. Students were asked to use their laptops to answer the VPs session independently.
2. **VPs Collaborative session (Group 2)**: A single session, in which VPs were conducted as a collaborative interactive activity within the lecture for 40 minutes. The VPs were projected on the classroom screen using a data show projector. The role of the tutor was to facilitate the session by asking why specific decisions were taken, what the consequence would be of choosing other options, providing feedback, and finally operating the VPs based on the decisions taken. During this session, students were allowed to go into a discussion with other students to reach the final consensus.

### Questioners

For the cognitive load, the study used the cognitive load questionnaire developed and validated by Leppink, Paas, van Gog, van der Vleuten, and van Merrienboer (2013) [17]. The language of the study is English, and the students are international students who are native speakers” so there was no need to change or modify the questioners. For measuring the intrinsic and extraneous cognitive load. It was an 8-item assessment, example of such items” The content of this lecture was very complex” & “the problem/s covered in this lecture was very complex”, the questionnaire was given at the end of each teaching session, and it was measured on a Likert scale from 1- to 10 [17].

The situational interest was a 6-item assessment which was validated before by another researcher Rotgans and Schmidt, 2017 [18], example of such items “I want to know more about this topic” & “I enjoy working on this topic” The situational interest questionnaire was given during the session every 10 minutes during conducting the different teaching sessions and it was measured on a Likert scale from 1 to 5. All the Questioners were answered on paper during face-to-face sessions.

### Statistical analysis

Data were collected and analyzed using SPSS software (IBM SPSS 27.0, SPSS Inc., Chicago, IL, USA). The data is considered ordinal data. Scores on each of the 6 SI and 8 Cognitive questions were summed for each participant, and the median was subsequently calculated for each group. After that, the median was compared using a non-parametric test (inferential tests). Friedman Repeated (ANOVA Test) and Wilcoxon signed-rank test (Pairwise comparison) were used for intragroup comparison. While Mann-Whiney U test (Pairwise comparison) and Kruskal Wallis test were used for intergroup comparison. In this study, the Significance level was set at *p* < .05.

## Results

Two dental cohorts have participated in the study, the first cohort includes 69 students (*n* = 69) and the second cohort includes 70 students (*n* = 70). All participants completed their tasks, so no one was excluded with a respondent rate of 100%. The average age is 18-21 years old and 35% male and 65% female.

### Situational interest – intragroup comparison

- **Group 1:** Comparing the situational interest scores at different time points during the lecture session showed no significant difference between the median score at repeated time points (Table 1). However, there was a significant difference between the median score at repeated time points during conducting the independent VPs session (Table 1).
- **Group 2:** Collaborative VPs session showed no significant difference between the median score at repeated time points (Table 1).

**Table 1:** Comparison of Situational interest median scores at different time points within Lecture, independent VPs, and Collaborative VPs sessions

Median Score-					***P*** value Friedman Repeated measure ANOVA Test
Type of Intervention	10 minutes	20 minutes	30 minutes	40 minutes	
**Lecture**	**22.00**	**22.00**	**21.5**	**22.00**	**0.727**
**Independent VPs**	**22.5**	**23.00**	**22.00**	**19.00**	**< 0.001**
**Collaborative VPs**	**23.5**	**23.00**	**24.00**	**24.00**	**0.051**
***P*** **value Kruskal Wallis Test**	**0.033**	**> 0.05**	**0.002**	**< 0.001**	

Following these results, the independent VPs session showed a negative median rank difference from 10 minutes to 20 minutes indicating that students showed high interest at the 10 minutes and this difference was significant (*p* value:0.012). The test showed also a positive median rank difference of situational interest from 20 minutes to 30 minutes. This means that the situational interest started to decline from 20 minutes to 30 minutes, but not significantly (*p*-value 0.116. The decline, however, was significant between 30 minutes and 40 minutes (*p*-value: < 0.001) (Table 2).

**Table 2:** Pairwise comparison is performed by Wilcoxon Signed Rank Test for independent VPs showing an increase in situational interest from 10 minutes to 20 minutes and a continuous decline to 40 minutes

Pairwise comparison Across selected two-time points		Median Difference	***p***-value (Wilcoxon signed-rank test)
**10**	**20**	**- 0.5**	**0.012**
	**30**	**0.5**	**0.001**
	**40**	**3.5**	**< 0.001**
**20**	**30**	**1**	**0.116**
	**40**	**4**	**< 0.001**
**30**	**40**	**3**	**< 0.001**

### Situational interest – intersessions comparison

**Fig. 1:**
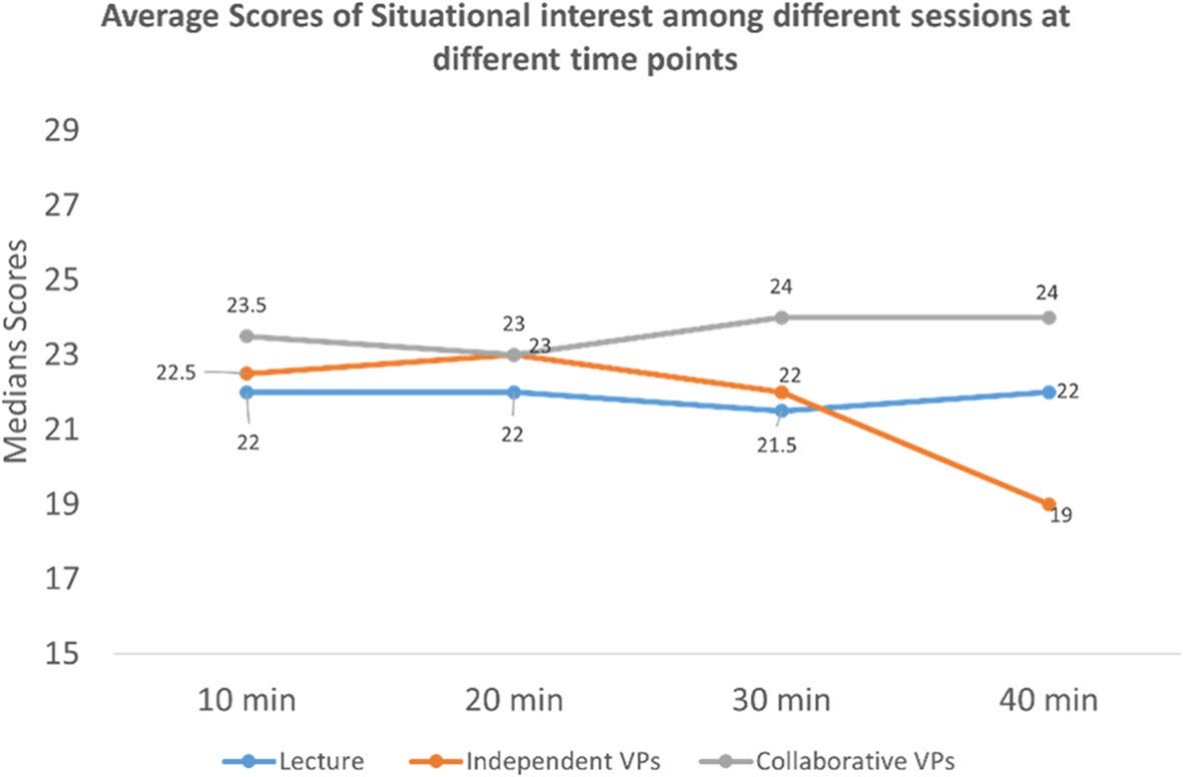
Average situational interest score among all the groups

**At 10 minutes**, the VPs’ collaborative session showed a high median score of situational interest than both lecture and independent VPs sessions, and the difference was significant (*p*-value: < 0.033). But no significant difference between a lecture and independent VPs sessions (Fig. 1).

**At 20 minutes**, there was no significant difference between all the sessions, although the lecture session showed a low median score of situational interest than both VPs independent and VPs collaborative sessions (Fig. 1).

**At 30 minutes,** the VPs collaborative session showed a high median score of situational interest than both lecture and VPs independent sessions, and the difference was significant (*p*-value: 0.002), and there was no significant difference between lecture and independent VPs sessions (Fig. 1).

**At 40 minutes,** the VPs interactive session showed an again high median score of situational interest than both the lecture and VPs independent session, and the difference was significant. However, the difference was also significant between the lecture and independent VPs sessions (*p*-value: < 0.001) (Fig. 1).

### Cognitive load

**Fig. 2:**
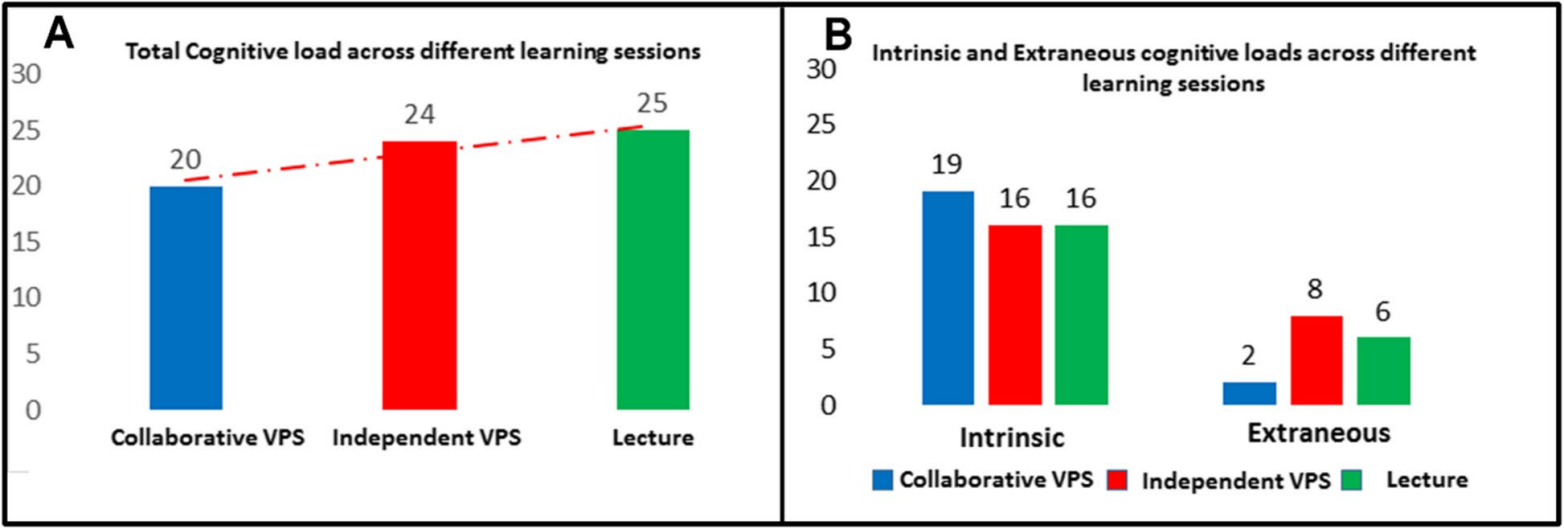
Comparison of total and different types of cognitive loads across different learning sessions. **A** is showing Total Cognitive Load across the learning sessions, and **B** is showing Intrinsic and Extraneous Cognitive loads across different learning sessions

The total cognitive load was found to be lowest in the VPs collaborative session, and highest in the lecture session (Fig. 2A). However, this difference was not significant. Intrinsic cognitive load was a little high in the VPs collaborative session than in both lecture and independent VPs sessions, however, this difference was not significant (Fig. 2B). The results also showed that the collaborative VPs session has the lowest extraneous cognitive load than the independent VPs and lecture sessions and this difference was a significant difference with *P*-value equal to 0.000 (*P* < 0.001).

## Discussion

Situational interest and cognitive load are considered two important factors that affect student engagement and motivation [13, 19]. Manipulation of both factors in a way that results in decreasing cognitive load and increasing situational interest enhances the engagement of the students.

Lecturing is considered the primary instructional method of teaching in higher education [20]. .It is a didactic teacher-centered method that is used mainly for large groups [20]. Although the lecture this format is often believed to be an effective method of teaching, evidence suggests lectures are relatively ineffective for motivating students [20, 21]. Our results showed that Situational interest scores remained stable throughout the activities for group 2, who did the VPs collaborative session. However, for group 1, the scores remained stable throughout the lecture activities but decreased at the end of the individual VPs session. These results suggested that situational interest is not immutable and can be triggered to enhance learners’ motivation and interest [22]. Many studies have found that the situational interest of students is increased when the teaching session is raising challenging situations and gaps of knowledge that prompt posing problems or raising questions (Knowledge deprivation theory) [11, 23–25]. Also, situational interest is increased when information and the content of the teaching session are relevant that continuously triggers leaner situational interest by helping them to make connections between learning material and real-life practice(trigger-maintenance theory) [23–25]. During VPs sessions, presenting information in form of a real clinical scenario helps the student to link and relate the new information to their clinical practice [26]. These facts might explain why in our study the situational interest was high in both VPs sessions than in the lecture based on the overall median sectors of each session.

Social interaction and peer collaboration and attention also were suggested to prompt and trigger situational interest [5, 27]. Those can be seen during collaborative VPs learning sessions but not much in lectures. This might also explain the low score of situational interest during the lecture.

Furthermore, several studies emphasized the importance of active mental engagement as an effective way of triggering the situational interest of students [11, 28, 29]. So, talking, writing, and elaborating during the learning session were suggested to enhance and trigger situational interest [29]. Also, designing instructional material in a way to assure maximum cognitive elements such as presenting novel challenging information, identifying gap of knowledge, and narratively organizing information will trigger situational interest [28, 30]. The nature of VPs as a learning tool offers more features of active mental engagement than lecture which may explain why students showed more situational interest than lecture.

Our study found that there is a significant difference between the situational interest at repeated time points during conducting the independent VPs after the lecture. For both lecture and independent VPs, the student showed high situational interest, in the beginning, this may be attributed to the fact that during this period most of the information is new for the student (novelty), and also a student in the beginning they identify and discover their gab of knowledge that they need to understand. It is well known that novelty and identifying gab of knowledge are the main factors that trigger the situational interest of the learners as they activate active mental engagement [10, 16, 28, 30]. However, lacking elaboration and peer discussion during lectures and independent VPs may be the reasons for the decline of situational interest that was detected. Or students’ “thirst” for knowledge was satisfied and their SI faded, as the knowledge-deprivation theory by Rotgans and Schmidt suggests it would [11]. Interestingly, it did not happen in the collaborative VPs session.

Situational interest is a short-lived psychological, cognitive and physiological status [31]. This concept can explain why the situational interest of the students was not stable during any teaching session. It was suggested that to maintain situational interest during a given learning session, a repeated triggering of a student’s interest is required [10, 16]. This can explain why situational interest was high in collaborative VPs sessions and maintained high along with different time points. Collaborative learning might explain in part the higher and steady levels of situational interest due to the collaborative learning and the discussion between the students among them-self and also with the instructor as suggested by the results of the literature explaining that peer discussion and collaborative learning drive the internal motivation of learning [5, 27, 32, 33].

Intrinsic cognitive load is mainly attributed to the complexity and difficulty of learning material [14]. In our study, the same topic was given over the three teaching modalities by the same instructor and was designed to be with the same difficulty and complexity. So, the intrinsic cognitive load is expected to be very close in all the sessions whether lecture, independent VPs, or collaborative VPs.

The collaborative VPs session showed the lowest extraneous cognitive load than the lecture and independent VPs sessions. These results are in agreement with previous studies explaining that lectures using PowerPoint containing a full amount of information, images, and even videos may lead to a high cognitive load [12]. It has been suggested that collaborative learning and social interaction through elaboration with peer discussion can reduce intrinsic and extraneous cognitive load [34]. Also, it was suggested that during collaborative VPs, students could link new information and new knowledge with the old or prior knowledge (schemata) or even build new schemata in a better way with less intrinsic and extraneous cognitive loads [5, 23–25, 35]. These studies attributed the efficiency of collaborative learning to several factors including a) the instructional design of VPs which containing simulated clinical scenario similar to the real practice, b) social discussion and elaboration with the peers and c) continuous feedback given to the student during the collaborative VPs session by the instructor.

## Conclusion

Our study suggested that using VPs in a collaborative learning activity enhances students’ situational interest with minimal investment in cognitive load than its use as an independent learning activity after the lecture.

### Limitations

Our study had certain limitations including a smaller number of VPs used, only one topic used in the study that can affect in situational interest or cognitive load of the learners according to their interest or their prior knowledge related to this topic and the study did not examine the effect of situational interest on learning outcomes (knowledge acquisition or retention) that can be suggested for a future study. Another limitation, is in group 1 students did the VPs session, they had already participated in the lecture about the same topic, therefore going into VPs with more knowledge than group 2 results in making comparisons between these scores are limited.

## Supplementary Information

**Additional file 1.**

**Additional file 2.**

## Data Availability

The datasets used and/or analyzed during the current study are available from the corresponding author upon reasonable request.
