# Supplementary material for: Effect of different teaching/learning approaches using virtual patients on student’s situational interest and cognitive load: a comparative study
Source: BMC Med Educ. 2022 Nov 7;22:763. doi: 10.1186/s12909-022-03831-8 (PMC9641945; doi:10.1186/s12909-022-03831-8)
Supplement: Supplementary file 1 — Additional file 1. [file 12909_2022_3831_MOESM1_ESM.docx]

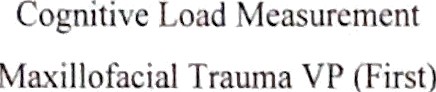


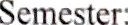


Student number:


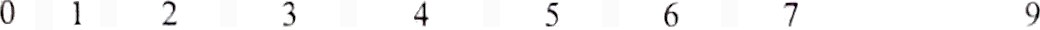
Please take your time to read cach ot the qucstlons care full; and respond to cach of lhe questions on thC Qfesented scale from 0 to 10, in u hich ’0' indicates mil at all the **case “NO”** and ' 10‘ indicates **completely** lhe **case** “Yes”:

| 1 . The ct›ntcnt o I this x irtual patient acti i ity « as x crj complex. | | | | | | | | | | |
| --- | --- | --- | --- | --- | --- | --- | --- | --- | --- | --- |
|  |  |  |  |  |  |  |  |  |  | I O |
| 2. The problem s cor ered in this › irtual patient acti› ity u as i crc › cv complex. | | | | | | | | | | |
| 0 | 1 | 2 | 3 | 4 | 5 | 6 | 7 | 8 | "I | 10 |
| 3. In this virtual paticnt activity, i ery complex terms w'cre mentioned. | | | | | | | | | | |
| 0 | 1 | 2 | 3 | 4 | 5 | 6 | 7 |  | 9 | 10 |
| 4. I invested a eg high mental eftort in the complexity of this virtual patielit activity. | | | | | | | | | | |
| 0 | 1 | 2 | 3 | 4 | 5 | 6 | 7 | 8 | 9 | 10 |
| 5. the explanations and instructions in this virtual patient activity u crc › eg unclear. | | | | | | | | | | |
| t) | 1 | 2 | 3 | 4 | 5 | G | 7 | 8 |  | 10 |
| 6. The explanations and instructions in this irtnal patient activity ere I nil of unclear language. | | | | | | | | | | |
| 0 | 1 | 2 | 3 | 4 | 3 | 5 | 7 | 8 | 9 | I fi |
| 7. The explanations and instructions in this virtual patient activity ii cre, in tenths of learning. i eg ineffective. | | | | | | | | | | |
| 0 | 1 | 2 | 3 | 4 | 5 | 6 | 7 | S | 9 | lo |
| 8. I inv’ested a very his h mental e1‘fort in unclear and incflcctii e explanations and  instructions in this virtual patient activity. | | | | | | | | | | |
| 0 | 1 | 2 | 3 | 4 | 5 | 6 | 7 | 8 | 9 | 10 |
