# Supplementary material for: Effect of different teaching/learning approaches using virtual patients on student’s situational interest and cognitive load: a comparative study
Source: BMC Med Educ. 2022 Nov 7;22:763. doi: 10.1186/s12909-022-03831-8 (PMC9641945; doi:10.1186/s12909-022-03831-8)
Supplement: Supplementary file 2 — Additional file 2. [file 12909_2022_3831_MOESM2_ESM.docx]

Situational Interest in Maxillofacial Trauma Lecture (40 mint)

Semester: 8 Student number:

- Now that you have a lecture about maxillofacial trauma, please indicate, on a scale from **(not true at all for me) to 5 (very true for me),** how true the Statements are for you right now.

1- I want to know more about this topic.

1 2 3 4 5

Not true Not true for me Neutral True for me Very true for me

at all

2- I enjoy working on this topic.

1 2 3 4 5

Not true Not true for me Neutral True for me Very true for me

at all

3- I think this topic is interesting.

1 2 3 4 5

Not true Not true for me Neutral True for me Very true for me

at all

4- I expect to master this topic well.

1 2 3 4 5

Not true Not true for me Neutral True for me Very true for me

at all

5- I am fully focused on this topic; I am not distracted by other things.

1 2 3 4 5

Not true Not true for me Neutral True for me Very true for me

at all

6- Presently, I feel bored.

1 2 3 4 5

Not true Not true for me Neutral True for me Very true for me

at all
